# Improvement of alkalophilicity of an alkaline xylanase Xyn11A-LC from *Bacillus* sp. SN5 by random mutation and Glu135 saturation mutagenesis

**DOI:** 10.1186/s12896-016-0310-9

**Published:** 2016-11-08

**Authors:** Wenqin Bai, Yufan Cao, Jun Liu, Qinhong Wang, Zhenhu Jia

**Affiliations:** 1 Department of Strategic and Integrative Research, Tianjin Institute of Industrial Biotechnology, Chinese Academy of Sciences, 300308 Tianjin, China; 2 College of Life Science, Shanxi Normal University, Linfen, 041004 China

**Keywords:** Xylanase, Alkalophilicity, Error-prone PCR, Site-directed mutation, Site-saturation mutagenesis, Catalytic efficiency

## Abstract

**Background:**

Family 11 alkaline xylanases have great potential economic applications in the pulp and paper industry. In this study, we would improve the alkalophilicity of family 11 alkaline xylanase Xyn11A-LC from *Bacillus* sp. SN5, for the better application in this field.

**Results:**

A random mutation library of Xyn11A-LC with about 10,000 clones was constructed by error-prone PCR. One mutant, M52-C10 (V116A and E135V), with improved alkalophilicity was obtained from the library. Site-directed mutation showed that the mutation E135V was responsible for the alkalophilicity of the mutant. The variant E135V shifted the optimum pH of the wild-type enzyme from 7.5 to 8.0. Compared to the relative activities of the wild type enzyme, those of the mutant E135V increased by 17.5, 18.9, 14.3 and 9.5 % at pH 8.5, 9.0, 9.5 and 10.0, respectively. Furthermore, Glu135 saturation mutagenesis showed that the only mutant to have better alkalophilicity than E135V was E135R. The optimal pH of the mutant E135R was 8.5, 1.0 pH units higher than that of the wild-type. In addition, compared to the wild-type enzyme, the mutations E135V and E135R increased the catalytic efficiency (*k*_cat_/*K*_m_) by 57 and 37 %, respectively. Structural analysis showed that the residue at position 135, located in the eight-residue loop on the protein surface, might improve the alkalophilicity and catalytic activity by the elimination of the negative charge and the formation of salt-bridge.

**Conclusions:**

Mutants E135V and E135R with improved alkalophilicity were obtained by directed evolution and site saturation mutagenesis. The residue at position 135 in the eight-residue loop on the protein surface was found to play an important role in the pH activity profile of family 11 xylanases. This study provided alkalophilic mutants for application in bleaching process, and it was also helpful to understand the alkaline adaptation mechanism of family 11 xylanases.

**Electronic supplementary material:**

The online version of this article (doi:10.1186/s12896-016-0310-9) contains supplementary material, which is available to authorized users.

## Background

Xylan is the second most abundant renewable resource in nature, following cellulose. It is composed of a backbone of β-1,4-linked D-xylopyranose residues, and its side chain is modified by different groups, such as acetate, D-glucuronate, 4-O-methyl-D-glucuronate, and α-L-arabinofuranose [1]. Because of its complex structure, the complete degradation of xylan requires the cooperative action of a variety of enzymes. Among them, endo-β-1, 4-xylanase (EC 3.2.1.8) is the crucial enzyme to randomly hydrolyze β-1,4-xylosidic linkages of xylan to xylooligosaccharides [2]. Based on hydrophobic cluster analysis and amino acid sequence similarities, xylanases are mainly classified into glycoside hydrolase (GH) families 10 and 11 [3]. Due to their ability to degrade xylan, xylanases have potential biotechnological applications in many fields [2]. A major application of xylanases is in the paper industry, in which the enzyme can reduce the amount of toxic chlorine-containing chemicals and improve the pulp brightness [4]. For example, pretreatment with Cartazyme HS-10 could reduce the chlorine consumption by 31 % at C-stage and 30 % reduction in total organochlorine content in the extraction stage effluent. The brightness was increased by 4.9 points in CEH sequence [5].

Family 11 xylanases can easily penetrate cellulose fiber networks without damaging the fibers because of their small molecular mass (about 20 kDa) and cellulase-free activity. Therefore, they are more suitable for the pulp bleaching process than family 10 enzymes [6]. Because the pulp bleaching process is usually in a high temperature (60–80 °C) and high pH (8–10) environment [2], the xylanases for this application are required to be thermophilic, thermostable, alkaliphilic, and alkali-stable [7, 8]. Although many xylanases have been cloned and characterized (http://www.cazy.org/GH11_characterized.html), few xylanases were found to be active and stable in the high temperature and alkaline condition. Therefore, engineering the native enzymes to achieve high activity and stability at high temperature and pH is in great demand.

Although many attempts on the modification of xylanases by rational design or directed evolution have been successful, such as increasing the optimal temperature or the catalytic activity of the enzyme [9–11], research on the improvement of alkalophilicity of the xylanase is limited [12]. Most of the mutations decreased the optimal pH of the enzyme [13, 14], or only shifted the optimum from acidity to neutrality rather than to the alkaline range [7, 15, 16]. Introducing excess arginine residues on the protein surface could increase the optimal pH of xylanase XynJ from aklaliphilic *Bacillus* sp. 41M-1 from 8.5 to 9.5 [17], but we could not replicate the result with the equivalent mutational change in the other xylanse in our previous study.

In the previous study, an alkaline xylanase Xyn11A-LC from *Bacillus* sp. SN5 was characterized [18]. It exhibited the highest catalytic activity at pH7.5, but little enzyme activity could be detected at pH 10.0. This property was not suitable for the pulp bleaching process. The three dimensional structure of xylanase Xyn11A-LC has been determined [19]. The molecular basis of alkaline adaptation of family 11 xylanase has been revealed [20]. These studies will contribute to engineer non-alkaline xylanase to function at a higher pH condition, but it is still hard to further improve the alkalophilicity of an alkaline xylanase by rational protein design. In this study, in order to obtain more alkaline mutants, a random mutation library of xylanase Xyn11A-LC was constructed by error-prone PCR. One mutant with improved alkalophilicity was obtained by a high-throughput screening system. Site-directed mutations and site-saturation mutagenesis were then carried out to validate the key role of the residue at position 135 on the pH activity profile of Xyn11A-LC. The mechanism of alkaline adaptation of the mutant was discussed by structural analysis.

## Results

### Construction and screening of random mutagenesis libraries

**Fig. 1:**
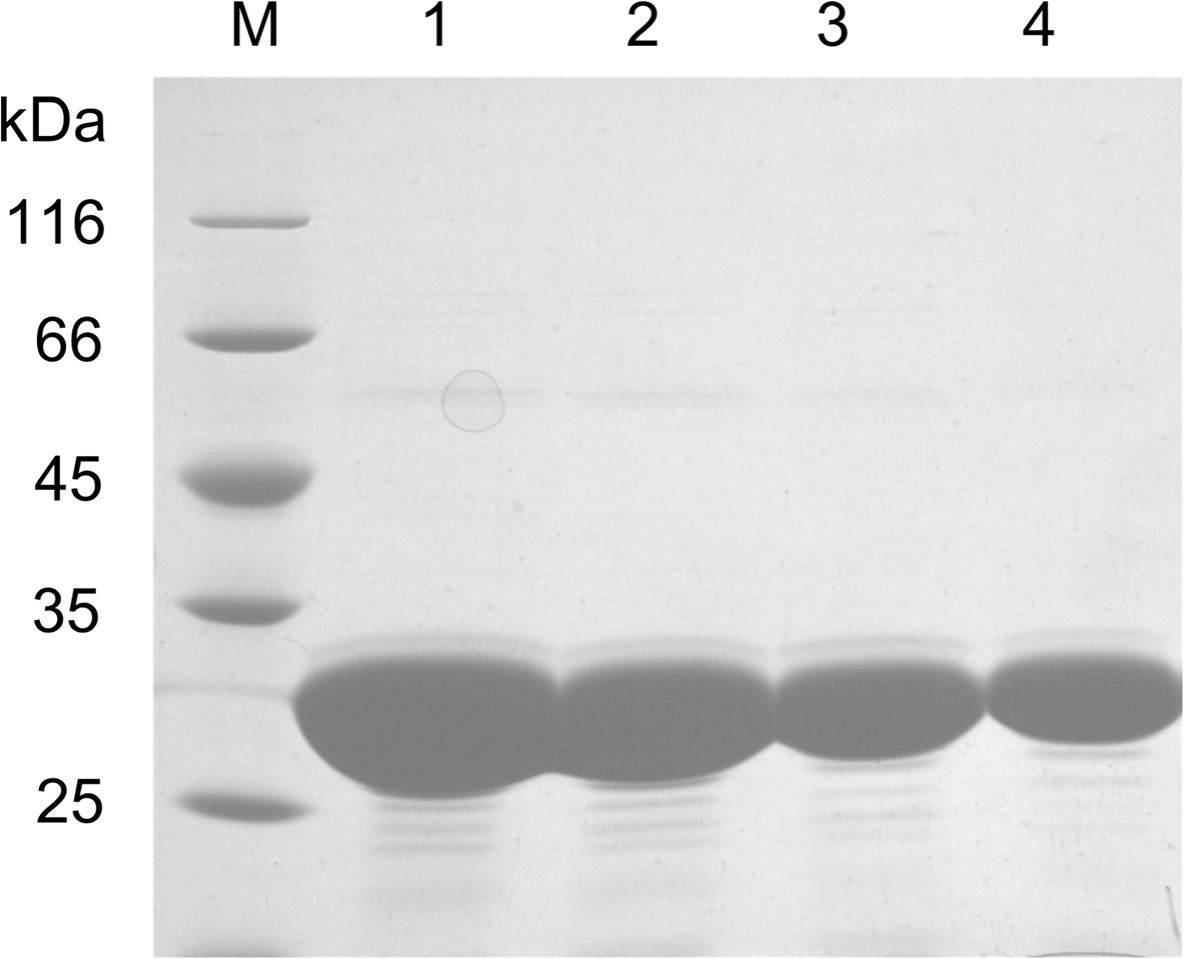
SDS–PAGE analysis of the purified xylanase Xyn11A-LC and mutants. Lane *M*, the protein molecular mass markers; Lane *1*–*4*, the purified recombinant Xyn11A-LC, M52-C10, E135V and E135R, respectively

Over 10,000 colonies were obtained from the random mutagenesis libraries. About 6000 transformants showing clear halos on the medium with Remazol Brilliant Blue xylan (RBB-xylan) were picked into 96-well plates for screening for the mutants with improved alkalophilicity. The selection criterion was an increase of at least 10 % in the pH 10/pH 7.5 activity ratio of the mutants compared to that of the wild type. For the second screening, a total of 54 mutants showing higher ratio than that of the wild type were obtained. Then, according to the pH activity profiles of crude enzymes of the wild type and 54 mutants, 8 mutants with improved alkalophilicity were obtained in the third screening. For the fourth screening, one mutant M52-C10 with improved alkalophilicity was obtained from above mentioned 8 mutants by determining pH activity profiles of the purified enzymes (Additional file 1: Figure S1). M52-C10 exhibited an optimal activity at pH 8.0 and corresponded to a basic shift of 0.5 pH units compared to the wild-type enzyme. Furthermore, the relative activities of mutant M52-C10 increased by 10, 15, 8 % at pH 8.5, 9.0 and 9.5, respectively (Fig. 1).

### Mutation site analysis

DNA sequencing showed that the mutant M52-C10 had two mutations, V116A and E135V. Site-directed mutagenesis revealed that only the E135V mutation showed the improvement of alkalophilicity (Fig. 1). The optimal pH of the mutant E135V increased to pH 8.0, which was 0.5 pH units higher than that of the wild-type enzyme. Compared to the relative activities of the wild type enzyme, those of the mutant E135V increased by 17.5, 18.9, 14.3 and 9.5 % at pH 8.5, 9.0, 9.5 and 10.0, respectively. Nevertheless, the pH activity profile of the mutant V116A was similar to that of the wild type enzyme. According to the above results, it could be concluded that Glu 135 might be one of the key residues involved in determining the pH activity profile of Xyn11A-LC.

**Fig. 2:**
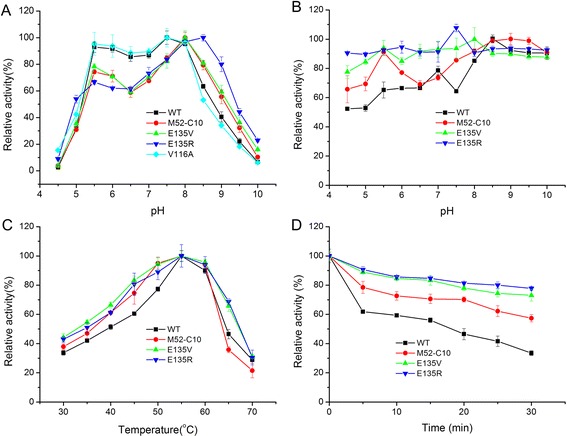
Effects of pH and temperature on the activity and stability of the recombinant Xyn11A-LC and mutants. **a** Effect of pH on the activity of Xyn11A-LC and mutants. The assay was performed in different pH buffer ranging from pH 4.5 to pH 10.0 at optimal temperature for 10 min. **b** Effect of pH on the stability of Xyn11A-LC and mutants. The enzyme was diluted in different buffer (pH 4.5-pH 10.0) at 37 °C for an hour, and the residual activities were measured at the optimal condition for 10 min. The activity of the purified enzyme without pre-incubation was set as 100 %. **c** Effect of temperature on the activity of Xyn11A-LC and mutants. The assay was performed at different temperature ranging from 30 to 70 °C for 10 min. **d** Effect of temperature on the stability of Xyn11A-LC and mutants. The purified enzymes were incubated in 50 mM Tris–HCl buffer (pH 8.0) without substrate for 30 min at 60 °C, respectively and taken out every 5 min. The residual xylanase activities were measured under the optimal condition for 10 min. The 0 min value was set as 100 %

In order to get more information about the influence of Glu 135 on the pH activity profile of Xyn11A-LC and to obtain more alkalophilic mutant, site-saturation mutagenesis was carried out by overlap extension PCR. The result showed that the only mutant to have better alkalophilicity than E135V was E135R. Its optimal pH was 8.5, 0.5 pH units higher than that of E135V. Mutations E135H, E135K, E135Q, E135M, E135Y and E135A exhibited maximal activity at pH 8.0, similar to the mutant E135V. The optimal pH of E135P was 6.0, 1.5 pH units lower than that of the wild-type. The ten other mutations exhibited maximal activity at pH 7.5, similar to the wild-type (Fig. 2).

### Characterization of the mutants and the wild type enzyme

To further elucidate the effects of Glu 135 site substitutions on enzyme characterization, other characterizations of the wild type and the mutants were determined. The mutants E135V and E135R were both stable over a broad pH range, retained over 80 % of original activity after incubation at 37 °C for 1 h over a pH range from 4.5 to 10.0, while the wild-type enzyme was stable only at alkaline pH from 8.0 to 10.0 (Fig. 2b). The optimal temperatures of the mutants M52-C10, E135V and E135R were all 55 °C, which was similar to that of the wild type. However, the mutants exhibited higher relative activity at the temperature range of 30–70 °C (Fig. 2c). In addition, the thermostability of the mutants was also determined. After incubation at 60 °C for 30 min, the mutants M52-C10, E135V and E135R retained 57.4, 73.1 and 77.8 % of their maximal activities, respectively, while the wild type enzyme only retained 33.5 % of its activity under the same conditions (Fig. 2d). This result showed that the displacement of the negative charge on the surface increased the thermostability of xylanase Xyn11A-LC.

The kinetic parameters of the wild type enzyme and the mutants toward beechwood xylan were determined under their optimal conditions. The *K*_m_, *V*_max_, and *k*_cat_ values of the enzyme were shown in Table 1. Compared with the wild type, the mutants M52-C10, E135V and E135R had lower *K*_m_ values, which indicated that the Glu 135 mutation increased the substrate affinity. Additionally, the *V*_max_ values and *k*_cat_ values of the mutant E135V and E135R are all higher than those of the wild type. Compared with the wild-type enzyme, the mutations E135V and E135R increased the catalytic efficiency (*k*_cat_/*K*_m_) by 57 and 37 %, respectively.

**Table 1:** The kinetic parameters of the wild-type enzyme and the mutants

Parameter	WT	M52-C10	E135V	E135R
*K*_m_ (mg ml^−1^)	3.3 ± 0.2	3.0 ± 0.4	2.5 ± 0.6	2.6 ± 0.5
*V*_max_ (μmol min^−1^ mg^−1^)	7178 ± 186	7355 ± 324	8546 ± 654	7734 ± 602
*k*_cat_ (s^−1^)	3230 ± 84	3310 ± 146	3846 ± 294	3480 ± 271
*k*_cat_ */K*_m_ (ml mg^−1^s^−1^)	978 ± 25	1103 ± 48	1538 ± 118	1339 ± 104

### Structural analyses of the mutants

**Fig. 3:**
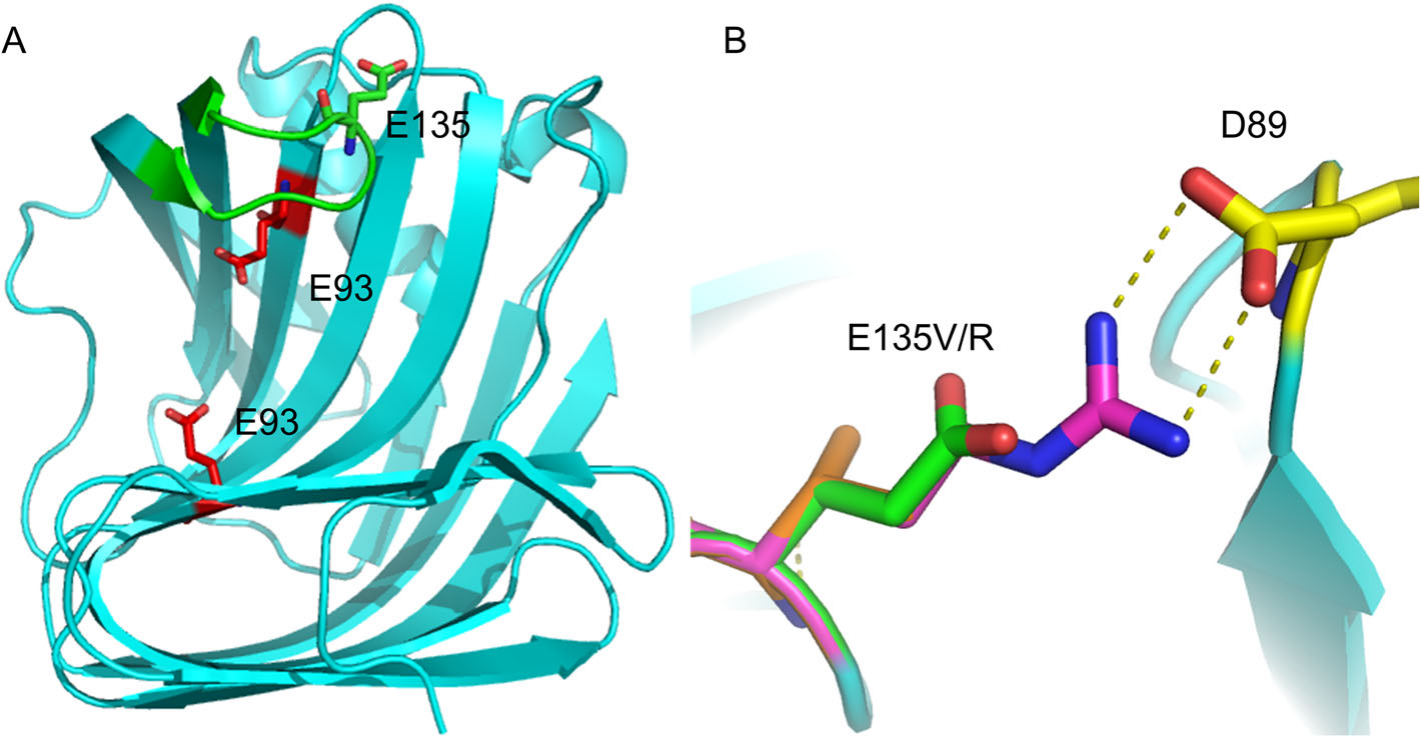
Locations and structural analysis of three key mutation sites. **a** Mutation location in the crystal structure of Xyn11A-LC (PDB accession no. 4IXL). The side chains of the catalytic residues (E93 and E183) and mutation (E135) are shown in *red* and *green*, respectively. A ‘thumb’ formed by the eight-residue loop is shown in *green*. **b** Location and mutation of E135V/R. E135, V135 and R135 are shown in *green*, *orange*, and *purple*, respectively. Salt bridges are represented in *yellow* by *dashed lines*

Some amino acid residues close to the catalytic residues (Glu 93 and Glu 183) are presumed to be responsible for the pH activity profile of Xyn11A-LC by changing the electrostatic potential of the active site [20]. However, the residue Glu 135 is far away from the two catalytic residues (Glu 93 and Glu 183). The distance between Glu 135 Oε1/Oε2 and Glu 93 Oε1/Oε2 is 12.12, 12.30, 13.22 and 13.23 Å, respectively, whereas the distance between Glu 135 Oε1/Oε2 and Glu 183 Oε1/Oε2 is 19.12, 19.60, 19.90 and 20.53 Å, respectively. Xyn11A-LC has the β-jelly roll structure typical of family 11 xylanases (Fig. 3a), which is also likened to the shape of a ‘right hand’ [21]. The eight-residue loop (Gln 131, Pro 132, Ser 133, Ile 134, Glu 135, Gly 136, Thr 137, and Ala 138) forms a ‘thumb’ that partly encloses the catalytic cleft. Glu 135 is located at the edge of the ‘thumb’ (Fig. 3a). The sequence and structure of the eight-residue loop of 13 family 11 xylanases, which were optimally active in acidic, neutral, or alkaline pH range, were compared, as shown in Table 2. The triad of residues that forms the tip of the thumb (Pro 132, Ser 133, and Ile 134) and Gly 136 are conserved in family 11 xylanase. The position 135 residue was a lysine residue in those xylanases with higher optimal pH, the corresponding residue is a glutamate or asparte residue in Xyn 11A-LC and some xylanases with lower optimal pH. In addition, the lysine residue in the eight-residue loop in higher alkaline xylanases was involved in the formation of a salt bridge (Table 2). It would be speculated that the elimination of the negative charge, the introduction of the positive charge, or the salt-bridge in the position 135 might be involved in the alkaline adaptation of xylanase.

**Table 2:** Sequence and structure comparison of the eight-residue loops of family 11 xylanases

Enzyme abbreviation	Source	PDB code	Eight-residue loop sequence	Salt-bridge in the eight-residue loop	Optimum pH
XynJ (∆XBD)	*Bacillus* sp. 41M-1	2DCK	QPSI**K**GTA	Lys135- Glu55	9 [17]
Xyl C	*alkalophilic Bacillus* (NCL 87-6-10)	2F6B	QPSI**K**GIA	Lys136-Glu56	8 [35]
BadX	*Bacillus agaradhaerens* AC13	1H4G	QPSI**K**GIA	Lys136- Glu56	8 [36]
Xyn11X	*Bacillus subtilis* B230	1IGO	QPSI**I**GIA	NA	8 [37]
Xyn11A-LC	*Bacillus* sp SN5	4IXL	QPSI**E**GTA	NA	7.5 [18]
XynA	*Bacillus subtilis* 168(1A1)	1XXN	APSI**D**GDR	NA	6–6.5 [38]
Xyl1	*Streptomyces* sp. S38	1HIX	APSV**E**GTK	NA	6 [39]
Bcx	*Bacillus circulans*	1XNB	APSI**D**GDR	NA	5.7 [40]
Xyn2	*Hypocrea jecorina* RUT-C30	1XYO	QPSI**I**GTA	NA	5.3 [21]
XYNI	*Trichoderma reesei*	1XYN	EPSI**Q**GTA	NA	3.5 [41]
XYL1	*Scytalidium acidophilum*	3M4F	EPSI**T**GTS	NA	3.2 [42]
XynA	*Aspergillus niger* CBS 513.88	1UKR	EPSI**T**GTS	NA	3 [43]
XynC	*Aspergillus kawachii*	1BK1	EPSI**T**GTS	NA	2 [44]

The key residues involved in the pH adaptation of enzymes are in bold and underlined

The mutation E135V with improved alkalophilicity might prove that the elimination of the negative charge at position 135 could contribute to the alkaline adaptation of xylanase. In order to validate the positive charge at position 135, the pH activity profiles of the mutations E135H, E135K and E135R were compared with E135V. The result showed that the pH activity profiles of the mutations E135H and E135K were similar to that of the E135V mutation, but E135R had higher alkapholicity than E135V, its optimal pH was increased to 8.5 (Fig. 2a). The structural analysis showed that a putative salt-bridge could be established between the introduced Arg 135 and Asp 89 because of a large guanidinium group of arginine, but it didn’t exist between Lys/His 135 and Asp 89 (Fig. 3b). The superior performance for Arg 135 relative to Lys 135 or His 135 suggests that the bidentate hydrogen bonding that is geometrically feasible for Arg 135 has more importance than simply the presence of a positive charge at this site.

## Discussion

Research on xylanase used in the paper industry has attracted increasing attention because it can reduce the cost, lower environmental pollution and improve the pulp quality. It requires the xylanase to be stable and active at high temperature and alkaline pH [2]. However, most of the xylanases are reported to be mesophilic or acidophilic enzymes. In this study, directed evolution of the enzyme was used by error-prone PCR. Xyn11A-LC was successfully engineered to improve its alkaline adaptation for potential industrial application for the pulp bleaching process.

The mutations E135V, E135K, E135H, E135A, E135Q, E135M, and E135Y all increased the optimal pH from 7.5 to 8.0. Furthermore, E135R increased the optimal pH from 7.5 to 8.5 (Fig. 2a). However, the optimal pH of E135P was 6.0, 1.5 pH units lower than that of the wild-type. It is speculated that a proline at position 135 is incompatible with the phi/psi for this beta turn, or it is likely to cause disruption through steric incompatibility with neighboring residues. Other studies also proved that the substitution of the residue in other xylanases corresponding to Glu 135 in xylanase Xyn11A-LC could also change the pH activity profile of enzymes. The E141A mutation in XYL1p from *Scytalidium acidophilum* increased the optimal pH from 3.2 to 4.0 [22]. The E141Q mutation in XynI from *Aureobasidium pullulans* changed the optimal pH from 2.5 to 3.5 [23]. The mutation E139K of Xyl1 of *Streptomyces* sp. S38 significantly raised the pH optimum from 6.0 to 7.5 [24]. Therefore, the position 135 site may play an important role in determining the pH activity profile of family 11 xylanase.

The residue Glu 135 located at the edge of the ‘thumb’ could also influence the catalytic activity of the enzyme. Two mutations (E135V and E135R) of xylanase Xyn11A-LC increased the catalytic activities to a different extent. The mutations E118A and E118Q of xylanase from *Aspergillus niger* for the same key position increased the activity by 50 and 16 %, respectively [25]. The same result was shown in XYL1p from *S. acidophilum* where E141A mutation elevated the specific activity by 50 % [22]. Furthermore, the importance of this residue in the catalytic activity of family 11 xylanases was confirmed by the inverse mutation, the mutation A139E in Xyl1 of *Streptomyces* sp. S38 decreased its specific activity by 75 % [26].

It was reported that the residues in the eight-residue loop were important in determining the pH optima of family 11 xylanase [7, 27]. The residue Glu 135 was located at the edge of the eight-residue loop on the protein surface (Fig. 3a). The substitution of Glu 135 residue with the valine residue might improve the alkalophilicity of the enzyme by eliminating the negative charge on the surface. The mutation E135R might further improve the alkalophilicity by introduction of arginines residues and a salt-bridge on the surface. The pH-dependent activity of the enzyme depends on the ionization of catalytic acid residues sensitive to the surface charge of the protein. Changing the surface charge of the enzyme could significantly shift the pH-activity profiles of the enzymes by changing the p*K*_a_ value of the active site, despite the two groups are far away from each other [28]. In our previous work, an increase in the number of the positive charge residues (Arg and Lys) and a decrease in the Glu residue on the protein surface were involved in the mechanism of alkaline adaptation of the family 11 xylanases, because the basic residues have high p*K*a values and can retain net positive charge even at high pH [20]. Several studies showed that increasing the number of arginines on the protein surface could improve the alkalophilicity of enzymes [17, 29]. In addition, there was a positive correlation between the number of salt bridges and alkaline adaptation of family 11 xylanases [20]. Other studies also indicated that the characteristic salt bridges in the catalytic cleft could contribute to the alkalophilicity of an alkaline xylanase XynJ from aklaliphilic *Bacillus* sp. 41M-1 [17, 30]. The salt bridge in the eight-residue loop might be beneficial to the stability of the enzyme in the alkaline condition and thus improve the alkalophilicity of the enzyme.

Research has indicated that the eight-residue loop possesses an opening and closing capability that modifies both the topology and the binding capacity of the active site, and the loop’s precise position determines the width of the catalytic cleft [27, 31]. Although, the residue Glu135 is far from the active site, it is located at the edge of the substrate binding cleft. The displacement of Glu 135 residue might eliminate the electrostatic repulsions between the cleft and the xylan which has a certain degree of negative charge because of glucuronic acid substituents [23]. This could increase the binding ability of the substrate and the cleft [23]. An intimate interaction with the substrate might protect the catalytic residues within the active site from the solvent and therefore allow the enzyme to function at a higher pH condition. It is also the possible cause of the decrease of the *K*_m_ values in the two mutations E135V and E135R. The position of the loop may be changed when a larger substrate binds in a slightly different conformation, which changes the distances between the catalytic residues and the substrate and thus affects the catalytic activity of the enzyme [32].

## Conclusion

Using directed evolution followed by site-directed mutagenesis, the residue at position 135 in Xyn11A-LC was speculated to be one of the key amino acids responsible for the pH activity profile of the enzyme. Furthermore, sequence alignment and structural analysis of family 11 xylanases with different optimum pH showed that the residue at position 135 located at the edge of the ‘thumb’ could affect the alkalophilicty by the elimination of the negative charge and the information of the salt-bridge. It will be useful to understand the alkaline adaptation of family 11 xylanases and engineer the enzyme to function at a higher pH condition. The mutant E135R is a promising and suitable candidate for paper pulp bio-bleaching.

## Methods

### Strains, plasmids, enzymes and reagents

The recombinant plasmid pET 28a-Xyn11A-LC was constructed by our research group [18]. *E.coli* BL21-Gold (DE3) strain was used as a host for the random mutagenesis library. *E.coli* BL21 (DE3) was used for protein production. GeneMorph II Random Mutagenesis Kit was purchased from Agilent technologies (USA). Beechwood xylan and RBB-xylan were purchased from Sigma (USA).

### Construction of random mutagenesis library

The random mutagenesis library was constructed by error-prone PCR using GeneMorph II Random Mutagenesis Kit according to the manual. The recombinant plasmid pET 28a-Xyn11A-LC was used as a template. In order to achieve medium mutation rate, initial target amount of template DNA is about 100 ng. The mutant gene was amplified by PCR using the primers ep-F and ep-R listed in Additional file 1: Table S1. The PCR cycling conditions were as follows: 2 min at 95 °C, then 30 cycles of 30 s at 95 °C, 30 s at 57 °C, and 30 s at 72 °C, followed by 10 min at 72 °C. The PCR products were purified using the gel extraction kit (Omega), digested with *Bam*HI and *Xho*I, and then ligated into the pET28a between *Bam*HI and *Xho*I sites. The resulting plasmids were transformed into *E.coli* BL21-Gold (DE3) competent cells by electro transformation and plated on LKX medium (Luria-Bertani agar plate, 50 μg/ml kanamycin, 0.2 % RBB-xylan, pH 9.0) [18].

### Screening for alkalophilic xylanase mutants

The colonies showing clear halos on above mentioned LKX medium were picked up and inoculated into 160 μl LBK medium (LB medium supplemented with 50 μg/ml kanamycin) in 96-well microplates for growth overnight at 37 °C with shaking at 900 rpm. Every mutant in the microplates was then replicated into fresh LBK medium containing 0.1 mM isopropyl β-D-1-thiogalactopyranoside (IPTG). After cultivation at 37 °C for 10 h, the cells were harvested by centrifugation at 4000 rpm at 4 °C for 10 min, re-suspended with lysis buffer (50 mM Tris–HCl buffer containing 10 mg/ml lysozyme, pH 8.0), and incubated at 37 °C for 60 min. The cell lysates were diluted 100-fold and used as the crude enzyme extract for downstream enzymatic reactions.

To screen the mutants with improved alkaline adaptation, xylanase activities were measured at pH 7.5 and pH 10.0, respectively. 10 μl of the crude enzyme extract was incubated with 100 μl 1 % beechwood xylan (*w/v*) dissolved in 50 mM Tris–HCl buffer (pH 7.5) and 50 mM Glycine–NaOH (pH 10.0), respectively. After incubation at 55 °C for 10 min, the released reducing sugars were assayed by the 3,5-dinitrosalicylic acid (DNS) method using xylose as the standard [33]. The variants with higher pH 10.0/pH 7.5 activity ratio than the wild type were considered as mutants with improved alkalophilicity. For the third screening, the mutants with significantly improved alkalophilicity were selected by detecting crude xylanase activity at pH from 7.0 to 10.0. For the fourth screening, the positive mutants were selected by determining the purified enzyme activity at pH from 4.5 to 10.0.

### Site-directed mutagenesis and site-saturation mutagenesis

Site-directed mutagenesis and site-saturation mutagenesis were performed using the overlap-extension PCR method with Phusion Hot Start II High-Fidelity DNA Polymerase as described [19]. The plasmid pET28a-Xyn11A-LC was used as the template. The primers containing mutated codons were listed in Additional file 1: Table S1. The PCR cycling conditions were listed as follows: 30 s at 98 °C, then 20 cycles of 30 s at 98 °C, 30 s at 55 °C, and 3 min at 72 °C, followed by 10 min at 72 °C. The amplification products were digested by *Dpn*I overnight at 37 °C and then transformed into *E. coli* BL21 (DE3) competent cells by electro transformation. The mutant xylanase gene from the transformants was verified by DNA sequencing performed by Genewiz BioSciences Company (Beijing, China).

### Expression and purification of enzymes

A single colony of strain BL21(DE3) harboring plasmid carrying gene of Xyn11A-LC or its mutant was grown in LBK medium at 37 °C until the OD600 reached 0.6–0.8. After a supplement with 1 mM IPTG, the culture was induced at 37 °C for 6 h. Cells were harvested by centrifugation at 8000 rpm for 10 min and disrupted by sonication in the lysis buffer (20 mM Tris–HCl, 500 mM NaCl, 10 mM imidazole, pH 8.0). Cell debris was removed by centrifugation at 16,000 rpm at 4 °C for 20 min. The supernatants were loaded on a nickel–nitrilotriacetic acid (Ni–NTA) column (Qiagen, Valencia, CA, USA) which was pre-equilibrated with the lysis buffer. Then the column was washed with washing buffer (20 mM Tris–HCl, 500 mM NaCl, 60 mM imidazole, pH8.0) and the bound protein was eluted with the elution buffer (50 mM Tris–HCl, 500 mM NaCl, 1 M imidazole, pH 8.0). Imidazole was removed by using a desalting column (GE Healthcare Bio-Sciences AB, Uppsala, Sweden) with 20 mM Tris–HCl buffer (pH 8.0). The purity of the proteins was analyzed by the sodium dodecyl sulfate polyacrylamide gel electrophoresis (SDS-PAGE). Protein concentrations were determined by a protein assay kit (Bio-Rad).

### Enzyme activity

The enzyme activities of the wild type Xyn11A-LC and its mutants were assayed by measuring the amount of reducing sugars from beechwood xylan by DNS method [33]. The reaction mixture containing 20 μl of 1 μg/ml xylanase solution and 480 μl of 1 % beechwood xylan (*w/v*) in different buffer was incubated in 55 °C for 10 min. After supplemented with 500 μl of DNS reagent, the mixture was boiled for 5 min and cooled to room temperature. Then the absorbance at 540 nm was determined with Spectra Max 190 microplate reader (Molecular Devices, Sunnyvale, CA, USA). One unit (U) of xylanase activity was defined as the amount of enzyme that released reducing sugar from xylan equivalent to 1 μmol xylose per minute under the assay condition. The assays were all performed in triplicate.

### pH activity profile and pH stability of the wild type and mutants

The effects of pH on the enzyme activity of the wild type Xyn11A-LC and its mutants was measured at 55 °C at pH from 4.5 to 10.0. The maximum value was set as 100 %. For pH stability, the purified xylanases were diluted to 2 μg/ml in different buffers with pH ranging from 4.5 to 10.0 and pre-incubated without substrate at 37 °C for 1 h. The residual activities were measured under optimal conditions. The enzyme activity of the purified xylanases without pre-incubation was set as 100 %. The buffers used were citrate-Na_2_HPO_4_ for pH 4.5–8.0, 50 mM boric acid/borate for pH 8.0–9.0, and 50 mM borate/NaOH for pH 9.5–10.0, respectively.

### Optimal temperature and thermostability

The effect of temperature on the xylanase activity was measured at optimal pH and a temperature range (30–70 °C). The maximum value was set as 100 %. For thermal stability, the purified xylanases were diluted to 2 μg/ml in 50 mM Tris–HCl buffer (pH 8.0), incubated at 60 °C and then taken out at an interval of 5 min. After incubation, the enzyme solution was immediately put in an ice bath for 10 min. The residual enzyme activities were assayed under optimal condition. The enzyme activity of purified protein without pre-incubation was set as 100 %.

### The kinetic parameters assay

To assay the kinetic parameters *K*_m_ and *V*_max_, reaction mixtures containing 1 μg/ml purified xylanases and different concentration of beechwood xylan (1–10 mg/ml) were incubated at optimum pH and temperature. The Michaelis-Menten parameters were calculated by nonlinear regression using Graphpad Prism 6.0 software (San Diego, CA, USA). The turn over number (*k*_cat_) was calculated based on the *V*_max_ value and the molecular weight of the targeted enzyme.

### Structural analysis

In our previous work, the structure of the wild type Xyn11A-LC was determined, and the atomic coordinates and structure factors have been submitted to the Protein Data Bank (PDB) under accession code 4IXL [19]. The coordinates of other twelve family 11 mesophilic xylanases with known pH activity profile were extracted from PDB. Multiple sequence alignment of the amino acid sequences was performed using the ClustalW program. The three dimensional structures of the mutants were modeled using Discovery Studio software. The surface residues of Xyn11A-LC with ˃30 % accessible surface areas were identified in Swiss-PDB Viewer using the default parameters [34]. Salt bridges were calculated online by submitting the coordinate files to website (http://bioinformatica.isa.cnr.it/ESBRI/); salt bridges were assigned when the distance between the two atoms of opposite charge was less than 4 Å. Structure visualization was performed using PyMOL software.

## Additional file

Additional file 1: Figure S1. Schematic diagram of a high-throughput screening of the positive mutant. **Table S1.** Primers used for random mutagenesis, site-directed mutagenesis and site saturation mutagenesis. (DOC 325 kb)

## Abbreviations

DNS: 3,5-dinitrosalicylic acid
IPTG: Isopropyl β-D-1-thiogalactoside
Ni–NTA: Nickel–nitrilotriacetic acid
PDB: Protein Data Bank
RBB-xylan: Remazol Brilliant Blue xylan
SDS-PAGE: Sodium dodecyl sulfate polyacrylamide gel electrophoresis
