# Supplementary material for: Improvement of alkalophilicity of an alkaline xylanase Xyn11A-LC from Bacillus sp. SN5 by random mutation and Glu135 saturation mutagenesis
Source: BMC Biotechnol. 2016 Nov 8;16:77. doi: 10.1186/s12896-016-0310-9 (PMC5101721; doi:10.1186/s12896-016-0310-9)
Supplement: Additional file 1: Figure S1. — Schematic diagram of a high-throughput screening of the positive mutant. Table S1. Primers used for random mutagenesis, site-directed mutagenesis and site saturation mutagenesis. (DOC 325 kb) [file 12896_2016_310_MOESM1_ESM.doc]

**Additional File**

Improvement of alkalophilicity of an alkaline xylanase Xyn11A-LC from *Bacillus* sp. SN5 by random mutation and Glu135 saturation mutagenesis

Wenqin Bai^1,2^*, Yufan Cao^1,2^, Jun Liu^1,2^, Qinhong Wang^1^, Zhenhu Jia^2^*,

^1^Department of Strategic and Integrative Research, Tianjin Institute of Industrial Biotechnology, Chinese Academy of Sciences, 300308 Tianjin, China.

^2^College of Life Science, Shanxi Normal University, Linfen 041004, China

**
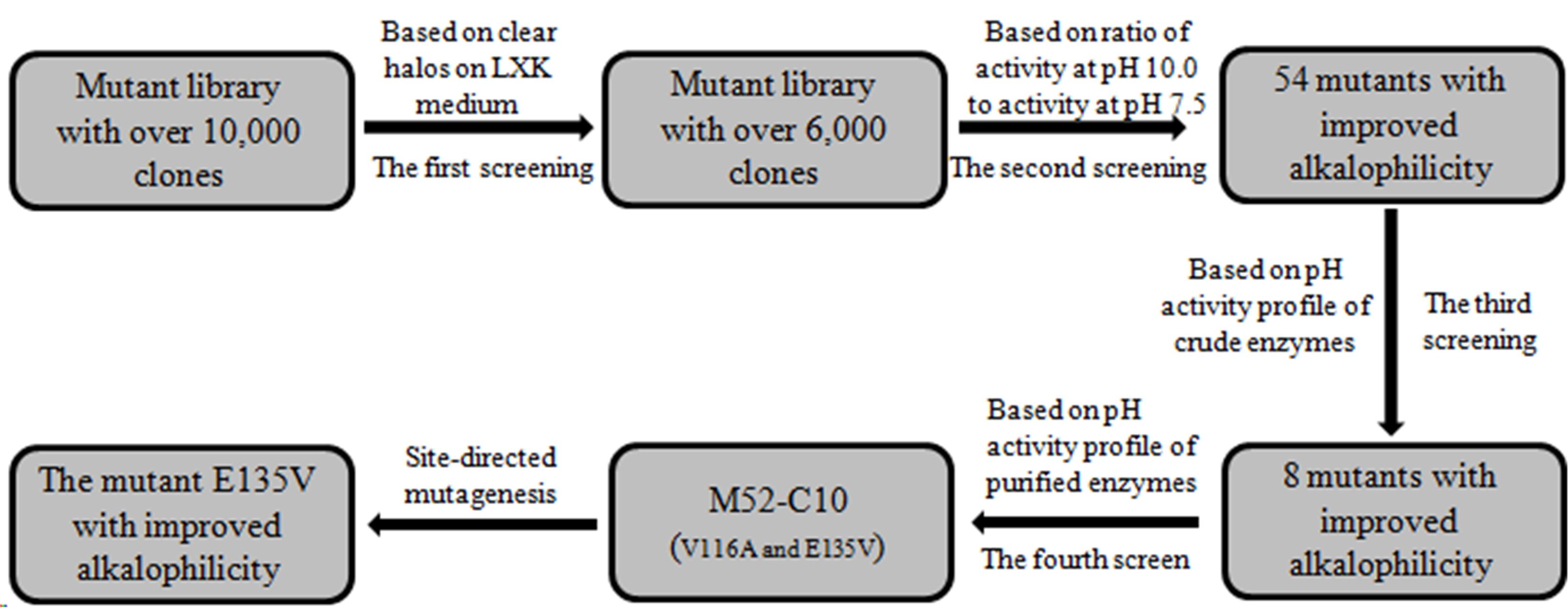
**

Figure S1 Schematic diagram of a high-throughput screening of the positive mutant

Table S1 Primers used for random mutagenesis, site-directed mutagenesis and site saturation mutagenesis

| Primers | Sequences |
| --- | --- |
| ep-F | 5'-GCATGGATCCCAAATCACTGGAAATGAAATCGG-3' |
| ep-R | 5'-GCTACTCGAGGCCGCCTATCGTTAAGTTATTTCG-3' |
| E135V-F | 5'-CAGCAGCCTTCCATT**GTT**GGAACTGCAACTTTC-3' |
| E135V-R | 5'-GAAAGTTGCAGTTCC**AAC**AATGGAAGGCTGCTG-3' |
| V116A-F | 5'-AAAGGCACTATTCAT**GCT**GATGGTGGAACTTAT-3' |
| V116A-R | 5'-ATAAGTTCCACCATC**AGC**ATGAATAGTGCCTTT-3' |
| E135K-F | 5'-CAGCAGCCTTCCATT**AAA**GGAACTGCAACTTTC-3' |
| E135K-R | 5'-GAAAGTTGCAGTTCC**TTT**AATGGAAGGCTGCTG-3' |
| E135R-F | 5'-CAGCAGCCTTCCATT**CGT**GGAACTGCAACTTTC-3' |
| E135R-R | 5'-GAAAGTTGCAGTTCC**ACG**AATGGAAGGCTGCTG-3' |
| E135H-F | 5'-CAGCAGCCTTCCATT**CAC**GGAACTGCAACTTTC-3' |
| E135H-R | 5'-GAAAGTTGCAGTTCC**GTG**AATGGAAGGCTGCTG-3' |
| E135D-F | 5'-CAGCAGCCTTCCATT**GAC**GGAACTGCAACTTTC-3' |
| E135D-R | 5'-GAAAGTTGCAGTTCC**GTC**AATGGAAGGCTGCTG-3' |
| E135Q-F | 5'-CAGCAGCCTTCCATT**CAG**GGAACTGCAACTTTC-3' |
| E135Q-R | 5'-GAAAGTTGCAGTTCC**CTG**AATGGAAGGCTGCTG-3' |
| E135N-F | 5'-CAGCAGCCTTCCATT**AAC**GGAACTGCAACTTTC-3' |
| E135N-R | 5'-GAAAGTTGCAGTTCC**GTT**AATGGAAGGCTGCTG-3' |
| E135A-F | 5'-CAGCAGCCTTCCATT**GCG**GGAACTGCAACTTTC-3' |
| E135A-R | 5'-GAAAGTTGCAGTTCC**CGC**AATGGAAGGCTGCTG-3' |
| E135G-F | 5'-CAGCAGCCTTCCATT**GGT**GGAACTGCAACTTTC-3' |
| E135G-R | 5'-GAAAGTTGCAGTTCC**ACC**AATGGAAGGCTGCTG-3' |
| E135S-F | 5'-CAGCAGCCTTCCATT**TCT**GGAACTGCAACTTTC-3' |
| E135S-R | 5'-GAAAGTTGCAGTTCC**AGA**AATGGAAGGCTGCTG-3' |
| E135T-F | 5'-CAGCAGCCTTCCATT**ACC**GGAACTGCAACTTTC-3' |
| E135T-R | 5'-GAAAGTTGCAGTTCC**GGT**AATGGAAGGCTGCTG-3' |
| E135C-F | 5'-CAGCAGCCTTCCATT**TGC**GGAACTGCAACTTTC-3' |
| E135C-R | 5'-GAAAGTTGCAGTTCC**GCA**AATGGAAGGCTGCTG-3' |
| E135M-F | 5'-CAGCAGCCTTCCATT**ATG**GGAACTGCAACTTTC-3' |
| E135M-R | 5'-GAAAGTTGCAGTTCC**CAT**AATGGAAGGCTGCTG-3' |
| E135P-F | 5'-CAGCAGCCTTCCATT**CCG**GGAACTGCAACTTTC-3' |
| E135P-R | 5'-GAAAGTTGCAGTTCC**CGG**AATGGAAGGCTGCTG-3' |
| E135Y-F | 5'-CAGCAGCCTTCCATT**TAC**GGAACTGCAACTTTC-3' |
| E135Y-R | 5'-GAAAGTTGCAGTTCC**GTA**AATGGAAGGCTGCTG-3' |
| E135F-F | 5'-CAGCAGCCTTCCATT**TTC**GGAACTGCAACTTTC-3' |
| E135F-R | 5'-GAAAGTTGCAGTTCC**GAA**AATGGAAGGCTGCTG-3' |
| E135W-F | 5'-CAGCAGCCTTCCATT**TGG**GGAACTGCAACTTTC-3' |
| E135W-R | 5'-GAAAGTTGCAGTTCC**CCA**AATGGAAGGCTGCTG-3' |
| E135L-F | 5'-CAGCAGCCTTCCATT**CTG**GGAACTGCAACTTTC-3' |
| E135L-R | 5'-GAAAGTTGCAGTTCC**CAG**AATGGAAGGCTGCTG-3' |
| E135I-F | 5'-CAGCAGCCTTCCATT**ATC**GGAACTGCAACTTTC-3' |
| E135I-R | 5'-GAAAGTTGCAGTTCC**GAT**AATGGAAGGCTGCTG-3' |

Restriction sites were underlined. Selected mutation sites were in bold
